# Medico-Chirurgical Transactions, Published by the Royal Medical and Chirurgical Society of London

**Published:** 1845-07

**Authors:** 


### ART. VI.

*Medico-Chirurgical Transactions, published by the Royal Medical and Chirurgical Society of London*, vol. XXVII.—*London*, 1844. 8vo, pp. 512.

THE volume recently issued from the halls of Berners street, is the bulkiest of which the annual contributions of the Medico-Chirurgical Society have yet required the production. Bulkiness is not the only quality of the tome; its general merit is remarkable; and though no single paper can be pointed to possessing a very high order of importance, all are more or less distinguished by the light they throw on points of scientific or practical interest. We shall notice them seriatim.

I. *An account of two cases of rupture of the ureter, or pelvis of the kidney, from external violence, followed by large effusion of urine into the abdomen;* by E. Stanley, President. A boy was squeezed between the wheel of a cart and a curb-stone, and brought into St. Bartholomew's Hospital, with severe contusion of the soft parts round the pelvis; the boy suffered from great pain in the lower part of the abdomen, and lay perfectly helpless. Nothing further is stated concerning the immediate consequences of the injury; but it appears that extensive subcutaneous suppuration subsequently occurred round the pelvis. By the end of the sixth week, recovery of the injured soft parts had considerably advanced. At this period the writer's attention was directed to a fulness, not before observed, on the right side of the abdomen. On further examination "a circumscribed, oblong swelling was recognized through the abdominal parietes, extending from the base of the chest downwards to within a short distance of Poupart's ligament; anteriorly, it terminated abruptly at the linea alba; posteriorly, it could be traced into the lumbar region, but it here presented no distinct boundary; the liver appeared to be pressed upwards by the swelling. . . . . Pressure on the swelling gave no pain, but a deep fluctuation in it could be recognized. The urine passed naturally, *as it had done throughout*, and that there was no distension of the bladder was ascertained by the introduction of a catheter." On puncture with a lancet a little clear yellow fluid escaped, in such manner as to show that it was "situated immediately beneath the abdominal muscles." Three weeks later the swelling grew more tense, and fifty-one ounces of a clear yellow fluid were discharged by puncture with a trochar. The application of leeches became necessary for the relief of pain, after each of the early punctures at least; the swelling was opened six times in all, and the boy discharged nine months after the accident, still exhibiting a swelling on the region mentioned, but one which had then for some time continued without increase or obvious diminution of size. The fluid was found on examination to contain urea, and was alkaline, highly albuminous, inodorous, of saline taste, and specific gravity 1008. It contained, besides urea, chloride of sodium, alkaline carbonates and sulphates, together with the peculiar extractive matter of urine. Mr. Taylor was of opinion that from the absence of mucus from the fluid, the communication with the urinary organs was not free, and was high up, as at the commencement of the ureter. Mr. Stanley thinks similarly, and appears to suppose (as is justifiable,) that the fluid had formed a cavity for itself by detaching the peritoneum from the abdominal and lumbar muscles.

In the other case, occurring in the practice of Mr. Vincent, death ensued in ten weeks after an injury much like that already described. The fluid had been withdrawn by puncture, but was pronounced by a "high chemical authority," (in one of his unanalytical moments, we presume,) not to be urine. On examination of the body a large cyst was found on the right side of the abdomen, behind the peritoneum, extending upwards to the diaphragm and downwards to the pelvis. A passage existed between the upper part of the cyst and the pelvis of the right kidney.

II. *Account of a case of cysticercus cellulosæ of the brain;* by Drewry Ottley, Esq. An emphysematous woman, aged 40, began to suffer in 1838, from giddiness and dull pain in the head; and had temporary numbness and loss of power in the right hand and arm, confusion of intellect and difficult articulation in July of that year. In 1839 she became subject to fits, with entire loss of consciousness and convulsions of the limbs. The attacks came on and ceased less suddenly than those of epilepsy; the convulsions came on as often as eight or ten times in as many hours, the stupor remaining in the intervals. In October 1840, she expired after frightful convulsions of twenty-four hours' duration. Post-mortem examination disclosed numerous small fibrous cysts in the pia mater of both hemispheres, they were most numerous on the left side. There were none in the white matter, the central ganglia, nor plexus choroides. The cerebral tissue round the cysts appeared natural. Each of the cysts was found occupied by a "vesicular worm, consisting of a posterior semi-transparent vesicle, and an anterior cylindrical portion which lay retracted in the former, like the finger of a glove turned inwards; the latter part was furnished at its extremity with a double circle of hooks, round which were seen four oval suckers." This case is interesting from the tolerably complete account of the symptoms given by the reporter. During the closing year of her life the patient suffered little from her pulmonary disease.

III. *On the cause of the occasional presence of spermatozoa in the fluid drawn from the sac of common hydrocele of the tunica vaginalis;* by J. Dalrymple. In the discussion which followed the reading of one of the papers, descriptive of the presence mentioned, it was suggested that possibly some of the seminiferous tubes had been punctured, in the operation for evacuating the fluid. And that this is not an improbable solution of the difficulty felt in accounting for the presence of these bodies, Mr. J. Dalrymple "attempts to explain."

Spermatozoa, the writer first observes, are of rare occurrence in the fluid of hydrocele, and are not to be confounded with vibriones generated in that fluid, if kept for some hours in a heated state of the atmosphere. It might appear sufficiently absurd to make the avoidance of this error a matter of serious caution, but in these days of microscopic mania, when even quacks appeal to the evidence of the instrument in favour of their charlatanism, the precaution appears to us superlatively wise.

Having, rather by inference than otherwise, (for Mr. Dalrymple appears in nowise covetous of drawing upon his head the wrath of certain embryo micrologists,*) disposed of some of the cases of *spermatozoa* found in the contents of the tunica vaginalis, the writer quotes from Scarpa and Le Dran statements regarding the altered position of the cord, the vas deferens and the epidydimis in scrotal hernia and hydrocele. We were unprepared to believe that surgeons are so generally unacquainted with these, as Mr. Dalrymple appears to hold; but admitting him to be correct in his notion, we are desirous of giving publicity to the account given of a dissection performed by himself. In a common hydrocele examined after death, and after inflation and drying, the appearances were as follow. "The body of the testicle was situated at the bottom, posterior and somewhat internal part of the cavity; and the vascular cord was seen running along the posterior wall of the sac, and emerging at the upper part, far behind the most prominent part of the swelling. The vas deferens, however, was situated quite external to this, and removed from the vascular cord nearly one inch. At the lower part it approached the testis, made a few of its larger coils, from which commenced the lower head of the epidydimis. As the latter body ascended again, it was still further separated, or dissected as it were, lying upon the lateral aspect of the sac, and distant from the body of the testicle, about an inch and a half; it continued to ascend, until it reached to nearly an inch and a quarter above the testis, when the position of parts was as follows: the testis and vascular cord at the most posterior part of the sac; the vas deferens next to it, but distant about half an inch: and lastly, the epidydimis still more external and anterior to the vas deferens. At the upper part, the epidydimis suddenly crossed the vas deferens, and descended in a curved line towards the body of the testicle, in which it finally merged." The description is illustrated by an engraving; and it appears from this, that the epidydimis and vas deferens are placed by no means out of danger of being punctured by the trochar.

IV. *Cases of carcinoma of the thyroid gland;* by Cæsar Hawkins, Esq. Mr. Hawkins having enhanced the importance of the cases, he is about to communicate, by reference to the idea of Scarpa that malignant disease of the thyroid gland only took place as a consequence of preceding alteration of adjacent parts, and by quoting the statement of Dr. Walshe, that "unless as a complication of preexisting encysted disease, or other form of bronchocele, cancer of the thyroid gland is singularly rare," proceeds to lay the particulars of those cases before his readers. In the first case, death did not ensue; the case therefore cannot be considered convincing, although several circumstances militate very strongly in favour of the accuracy of Mr. Hawkins's diagnosis. But the following history terminating by the post-mortem examination is particularly valuable.

A man, aged 50, was admitted into St. George's Hospital, having the general aspect of perfect health, with a considerable enlargement of the whole thyroid gland, but particularly of the right lobe. The tumour was smooth, very firm and solid; the skin unattached and unaltered in colour; the superficial veins large. The larynx and trachea were thrown very

* The bitterness and envious spirit of physiologists have long been proverbial; but the micrological section of those philosophers bid fair to acquire a special reputation for amenity of temper altogether beyond the chance of rivalry.

much to the left side; but inspiration was more easily performed than deglutition. The tumour was free from pain or tenderness; and had made its first appearance five weeks before his admission. The man lost flesh; and died about two months after he came under observation; the whole course of the disease was consequently not more than fourteen weeks. Dysphagia appears to have been constant, but sometimes in addition, paroxysmal; vomiting sometimes occurred regularly at a certain period after eating, and at other times took place violently during his meal. The patient suffered much from pain in the epigastrium and hypochondria, and had tenderness over the stomach. He began to vomit some coagula of blood a week or so before death.

The right internal jugular vein, common carotid artery, and pneumogastric nerve were separated from each other by the pressure of the morbid growth. The coats of the vein had in one place been absorbed, and some of the tumour protruded into its anterior. The vagus nerve was flattened and its fibrils separated. The tumour occupied almost the entire of the thyroid gland, and destroyed the anterior wall of the œsophagus, protruding into its interior in the form of a large ulcerated mass; below the cricoid cartilage, a large ulcerated opening led into the trachea. The tumour presented the structure of genuine scirrhus; and there were many small encephaloid tubercles at the base of both lungs, and in the cellular tissue under the costal pleura. The stomach was perfectly healthy.

The epigastric pain and tenderness, the vomiting, and latterly, hematemesis led Mr. Hawkins into the very excusable error of supposing that the stomach was cancerously diseased. The difficulty of swallowing, and the vomiting are otherwise and readily to be accounted for by the post-mortem appearances in the œsophagus. But how may the epigastric suffering be explained, as the stomach was sound? Plausibly enough, with Mr. Hawkins, by the expanded and flattened state of the vagus nerve.

We are at a loss to determine why Mr. Hawkins considers it justifiable to regard the cancerous growths existing in the thyroid gland in a case published by Velpeau as encephaloid, while the narrator of the case affirms they were scirrhous, and describes certain of the characters peculiar to the latter kind of formation. Mr. Hawkins objects to the alleged statement of Dr. Walshe that cancer of the thyroid gland is generally of the scirrhous species. But (without meaning to give the weight of our opinion to either side,) we may observe that on referring to Dr. Walshe's Essay we find he makes the statement only in reference to cancerous deposition complicating preexisting disease, and not to cancer in general.

Mr. Hawkins next gives a very brief outline of a case of medullary disease of the gland; here the characters of fungus hæmatodes appear to have been well marked.

As an appendix to this paper appears a case of scirrhus of the thyroid gland by Mr. Brown. The subject of this case was a male, aged 60, who began in December 1842, to suffer from uneasiness about the larynx, slight cough and hoarseness. A hard swelling presented itself in the situation of the left lobe of the thyroid gland, apparently extending internally, and pressing on the œsophagus, as there was great difficulty experienced in swallowing. The integuments in the neighbourhood became thickly studded with cancerous tubercles. Violent, convulsive, and almost incessant cough, with copious muco-purulent expectoration, streaked with scarlet blood, was one of the most prominent symptoms. The patient died in a state of very considerable emaciation in June 1843.

Numerous hard tubercles were seated in the skin of the throat, chest, and abdomen, composed of the same substance as the main diseased mass which occupied the left lobe of the thyroid gland. This lobe was somewhat enlarged and converted into a mass of carcinomatous structure, white, hard as cartilage, and with some gritty particles dispersed through it.

V. An account of an *Alarming syncope from the admission of air into a vein during an amputation at the shoulder-joint;* by Bransby Cooper, Esq. A woman aged 19, had her arm amputated at the shoulder-joint for malignant disease of the humerus. While the operator was dissecting an enlarged gland from the axilla, he distinctly heard a gurgling noise, like air escaping from a narrow-necked bottle, and at the same instant the patient fell into a state of collapse from which she was fortunately roused, and after an hour's assiduous care, removed from the theatre. Beyond the mere fact of the patient's life being saved, (and the case does not stand alone in this respect,) there is nothing remarkable in these circumstances; they are the ordinary symptoms of entry of air into the veins. But more interesting phenomena are noticed afterwards. While reaction advanced, she "maintained a constant motion of alternate flexion and extension of the right leg, while the left remained perfectly quiescent. She continually complained of pain extending up the right side of the head and neck." When placed in bed she passed her feces and urine involuntarily. This action of the right leg continued for the following six or seven days; on one occasion there were involuntary flexions of the left, which she had not the power of extending. On the twenty-fifth day after the operation, the woman was able to sit up in a chair and take her dinner. She then complained of great numbness and loss of power in the left leg, which she dragged after her; it was as sensitive as the right. On the fortieth day she was discharged from the hospital, having no other unfavorable symptom than some slight dragging of the limb; and about five months after was readmitted with a tumour (of the same kind as the humeral) in the left scapula. After having experienced for a day or two great pain in the lower extremities; they became paralysed, and she suffered at first from inability to void her urine, and afterwards incontinence of this, with torpor of the bowels. She died two months after readmission. A large fungoid mass appeared in the right side of the chest. The tumour of the scapula, "extended to the posterior part of the vertebral column, adhered principally to the second dorsal vertebra, the arch of which was pressed inwards, so as to encroach upon the vertebral canal at that part. The medulla appeared quite healthy, although it must have been slightly compressed by the vertebral arch." The brain and viscera were perfectly healthy.

VI. *Account of a horn developed from the human skin; with observations on the pathology of certain disorders of the sebaceous glands;* by Mr. Erasmus Wilson. In certain cases the sebaceous substance acquires an abnormal degree of density. The substance, accumulated in undue quantity and impacted, exerts much pressure on the walls of the follicle, an amount of pressure sufficient, in the language of Mr. Wilson, to "abrogate" the special function of that organ. The formation of epithelium, however, still continues, whereby a mass in layers is produced, sometimes of considerable size. The aperture of the follicle remains open, and is more or less distended in proportion to the extent of the tumour; but from the nature of the collection there is no tendency to its escape. The laminated texture and epidermal composition of these collections, constitute their distinctive character; the peculiar materials of sebaceous secretion being absent. If now the upper wall of the laminated collection be removed, and this consequently exposed to the influence of the atmosphere, any retained moisture becomes evaporated, and the mass hardens, in other words is "converted into horn." Fresh layers go on accumulating from below, the indurated mass is forced outwards, increases in size, and will continue to increase for years, unless interfered with by surgical operation. So are produced horns.

Mr. Wilson proceeds to describe a horn removed from the skin of an old woman; and is minute and satisfactory in his description. Persons interested in these *nugæ* of pathology will find the writer's details instructive.

VII. *On the early organization of coagula and mixed fibrinous effusions, under certain conditions of the system;* by John Dalrymple. This subject is not a new one to Mr. Dalrymple; he was the describer some years ago of Mr. Busk's injection of a clot contained between the surface of the tibia and its periosteum. We have examined portions of this clot, and regard the vessels discernible in it, as unquestionably of new formation; the notion that the injected canals were the original vessels between the periosteum and bone, stretched and separated by the clot, is shown by the form, mode of arrangement, and general characters of those canals to be perfectly inadmissible.

But a more important question remains, and one not so readily answerable. This question is whether the newly vascularized substance was simply extravasated blood, or whether in addition to extravasated blood there was present some effused liquor sanguinis or lymph. Under the former circumstances it must be admitted that the blood becomes in proper substance vascularized; and in the latter we have simply proof that the pressure of the colouring matter, &c. of the blood will not prevent lymph, with which it chances to be associated, from going through the usual changes of advancing development. The case of Messrs. Busk and Dalrymple does not satisfactorily remove this difficulty.

Mr. Dalrymple appears to have, of late at least, felt this. A Lascar died with scurvy on board the Dreadnought; in his knee-joint were found many coagula, some adherent to the reflected synovial membrane surrounding the cartilages of the femur and tibia, and some loose in the cavity of the joint. The limb having been very successfully injected by Mr. Busk, the attached coagula were found to be permeated with new and numerous capillary vessels. Mr. Dalrymple thought this a good opportunity for confirming his previous results, and not content with the mere fact of the clot having been injected, proceeded to a microscopical examination of the morbid parts, before they had been altered by immersion in spirits of wine. The colour of the masses was found to depend upon red blood-corpuscles in an entire state mingled with fibrinous globules. The firmness of these clots was due to the "advancing organization of the fibrine itself, the fibrinous cells being found in all stages, from the granulated sphere to the caudate cell, ultimately developing into filamentous tissue." That is, the series from the exudation-corpuscle at one end to the wavy filament at the other, of the progressive steps of this re-formation were observed. But in what manner or mode does the discovery of this cell-development throw light on the question, which we stated was, in the present state of knowledge, unanswered and unanswerable? None in the world. Mr. Dalrymple leaves the matter just as he found it.

There were evidences of advance towards the formation of structure in some of the loose coagula, and at a period when "it is obvious no new vessel could have formed within the mass." This is a point that requires extended observation, adds the writer, and "may have some connexion with the obscure subject of the production of loose cartilages, sometimes found in joints and bursal cavities." It not only may, but must have such connexion, and has already been examined under this point of view by various persons.

"It is not, however, now contended," concludes Mr. Dalrymple, "nor was it in my former paper, that *ordinary extravasations of blood in the healthy body* become organized, because the vitality of the surrounding parts is higher than that of the blood so effused, and the absorbents under such conditions, effect their ordinary changes in consequence of a tendency to disintegration, rather than to an advanced development of the effused blood."

VIII. *A case of extirpation of an ovarian cyst, terminating fatally;* by Mr. Bransby Cooper. A healthy looking woman, aged 32, who had always menstruated irregularly, observed enlargement of the abdomen five years before the operation, one year before marriage: (she had never become pregnant)—the swelling twice disappeared through diuresis; and the woman was besides tapped twice. She had never suffered from symptoms justifying the notion that peritonitis had at any time occurred. Her health being excellent, she appeared an admirable subject for operation. This was performed as follows: "An incision was made below the umbilicus in the median line, between three and four inches in length; and the peritoneal cavity being opened to a small extent, a little ascitic fluid escaped. The finger was introduced and passed around the opening, and a few slight adhesions broken down. An incision was now made through the integuments, commencing three inches below the ensiform cartilage, and extending to the upper part of the first incision, carefully avoiding the umbilicus; and the subcutaneous structures were then divided by a probe-pointed bistoury, cutting from below upwards, the finger being used as a director. The wound was also enlarged towards the pubis." There was very little hemorrhage. A broad and thin pedicle connected with the right ovary soon came into view, which was tied and the mass removed; it weighed thirty-two pounds, it was mainly composed of a single cyst, but there was a small collection of compound cysts in one part. The nature of the mass was consequently the most favorable to success; there were no peritoneal adhesions, the woman was in excellent health;—and she died from peritonitis on the fifth day.

IX. The *Removal of a diseased ovarium, terminating fatally on the seventh day after the operation;* by Mr. Greenhow. This woman was obviously not in so favorable a condition for operation as the subject submitted to the knife by Mr. Bransby Cooper. She "had no symptoms of constitutional disorder," however, when operated upon, "the tongue was clean, the appetite good, the bowels regular, the urine free and natural, and the patient able to sit up three hours in the day; the pulse seventy-six, soft; occasional œdema of the legs had occurred. No pain nor tenderness in any part of the abdomen, except at one point towards the right iliac region, where the original moveable swelling was first felt." The incision made reached from a little below the ensiform cartilage to near the pubis; several adhesions existed in different parts of the tumour. The operation was well borne, but vomiting occurred several times towards the close. The quantity of blood lost did not exceed six ounces. This woman had peritonitis, and died on the seventh day,—death being possibly hastened by disease of the pyloric end of the stomach.

The narrators of these cases of failure deserve the approbation of all honest men.

X. *On the state in which the uric acid exists in the urine;* by H. B. Jones, M.A. Dr. Jones commences the narration of his attempt to cross this *pons asinorum* of ordinary chemists by a most laconic statement of the efforts, in the same direction, of Berzelius, Prout, Simon, and Becquerel. Why those of Donné, Vigla, Quevenne, Guibourt and Rayer should receive no mention at his hands does not very clearly appear.

Dr. Jones next turns to the description of the appearance and properties of the deposit containing uric acid which may be obtained from urine by evaporation under the air-pump. This deposit consists of a most minute globular powder, the particles of which are amorphous, exhibit particular motions, and after solution in water are redeposited on cooling with the same character as before. Any acid changes the powder into angular crystals of uric acid. The entire paper is very creditable to its author; but will scarcely bear analysis.

XI. A *Case of extensive carcinoma of the lungs, with some practical remarks;* by Dr. Burrows. The subject of this case was a female, aged 20, delivered of her first child three months after the first occurrence of pulmonary symptoms, and three months before admission into St. Bartholomew's Hospital. A month before the latter event, she had hemoptysis. On admission the main symptoms were as follow: fulness and œdema of the face, lividity of the lips; respiration 40; pulse 132 (rising to 160 in the sitting position;) pain between the shoulders and at the epigastrium; dyspnea; paroxysms of cough; sputa scanty, intimately blended with blood and of a uniform pink colour, resembling currant juice; lymphatic glands of right side of neck swollen, hard and tender; cervical veins distended; clear exaggerated respiration with increased resonance on percussion audible all over the left lung. On the right side of the chest diminished resonance on percussion in the upper part, perfect dulness below the third rib in front and beneath the spine of the scapula, entirely down to the right hypochrondrium; feeble respiration with prolonged expiration in upper part of right lung; similar respiration along the right side of the spine; bronchial respiration with chirping sounds beneath the mammæ and in the lower axillary region. The weakness of the voice prevented vocal resonance and vibration from furnishing any signs; the heart's sounds were natural and not transmitted beyond the precordial region. Upon these phenomena Dr. Burrows based the diagnosis of malignant disease of the lung, and the following passage contains the reasons for the opinion: "the extreme dulness on percussion, the absence of all vocal phenomena, and the constant decubitus on the right side, seemed to support the supposition of pleuritic effusion. But opposed to this conclusion were the presence of bronchial respiratory murmur in the axilla and hypochondrium, when the dulness on percussion was greatest, and the absence of œgophony and of loud bronchial breathing and bronchophony along the right of the spinal column. There were also present physical signs which, when taken together, were inconsistent with consolidation of the right lung, viz., the extreme dulness on percussion, the very feeble bronchial respiratory murmur, the absence of bronchophony or vocal vibrations, and the want of transmission of the heart's sounds to the right of the sternum. Hence the diagnosis of some anomalous disease of the right lung was formed; and when the peculiar aspect of the woman, the distressing dyspnea and cough, with the singular currant-juice sputa, the enlarged cervical glands and the history of the case, unlike that of pneumonia or pleurisy, were all duly considered, the opinion was hazarded that she was suffering from malignant formations in the right lung." The woman died fifteen days after admission. A cerebriform mass of considerable magnitude projected from the middle lobe of the right lung; towards the root of this was a similar mass, and the middle lobe resembled both in appearance. The superficial parts of the lower lobe were converted into a darkish brown friable substance, studded with white points; portions of the tumour had protruded into the interior of the main right bronchus; the right pleura contained four pints of an olive brown serum; the left pleura and lung exhibited nothing remarkable; the right pulmonary vein was compressed, obliterated, or perforated by the morbid masses.

Dr. Burrows insists on the importance of correct diagnosis in such cases as the present, as a means of preventing the physician attempting too much in the way of treatment, and in fact shortening the patient's existence by the application of remedies fitted only for very different diseases. He then having briefly traced the progress of knowledge on this subject in the writings of Laennec, Andral, Louis, Stokes, and Walshe, proceeds to compare the general symptoms and physical signs manifested in his own case with those described by the best authorities. Having ourselves devoted some considerable space to an article on this subject (April 1843,) for his courteous mention of which we are beholden to Dr. Burrows, we feel it unnecessary to dwell further upon it on the present occasion, especially as the author's remarks, however judicious, are not actually novel.

XII. *An account of certain cases of acute disease in the throat and larynx, one of which was cured by tracheotomy,* follows, from the pen of Dr. J. A. Wilson. Dr. Wilson having seen two persons die thirteen years ago of inflammation, with thickening and purulent infiltration of the neighbourhood of the chordæ vocales, (which inflammation had not extended below the glottis, and which persons might consequently, he presumes, have been saved by tracheotomy,) found the experience thus acquired extremely useful in the month of July 1843. He was called to a person labouring under symptoms of intense laryngitis, which leeching, calomel, and other active measures were found incapable of controlling. Twenty-four ounces of blood were taken from the arm. Suffocation became imminent, and the trachea was at once divided beneath the thyroid gland. Recovery followed. The case is a most instructive one; and in the tenor of the following remarks we completely concur: "For the operation in this disease, practically, it is never too late; but the chance of its success is greatly lessened by a delay, under which the patient, poisoned by his own blood, loses consciousness, and becomes convulsed. Like the ligature of a bleeding artery, tracheotomy is ruled, without consultation, in all cases where danger is imminent, and within a range of seconds or minutes, might sometimes be justfied even after apparent death." The curved trochar, if at hand, is certainly the best instrument for opening the trachea with.

Another case follows, in which the operation was equally called for and equally successful.

XIII. *On the presence of oxalate of lime in the urine;* by H. B. Jones, M.A. The states of the system in which the octohedral crystals, that are considered to be composed of oxalate of lime, present themselves in the urine are exceedingly variable. Dr. Jones has found such crystals appear in acute rheumatism and gout, chronic rheumatism, aggravated hypochondriasis, hysteria, and diabetes. Donné and Rayer had noticed a connexion between spermatorrhea and the occurrence of these crystals in the urine. Dr. Jones has confirmed their observations.

Dr. Jones feeling desirous of a further demonstration of the nature of these crystals, than that afforded by the microscope, availed himself of the opportunity of chemical examination furnished in a case of rheumatism when the octohedra were exceedingly abundant:

"The urinary sediment was thrown on a filter and washed with distilled water. The red residue was dried, reduced to a fine powder, and treated with dilute hydrochloric acid, which left most of the uric acid undissolved. The acid liquid was filtered, and ammonia gave a very considerable precipitate, when added in excess. When evaporated to dryness, and heated on platinum, the muriate of ammonia was driven off, and the residue effervesced strongly, when thrown into dilute acid, and left an alkaline ash, when heated more highly. The ash was with difficulty soluble in water, and gave a precipitate with oxalate of ammonia. Hence some organic acid salt of lime was present; and as oxalate of lime is known to occur in octohedral crystals, the conclusion that these crystals were oxalate of lime is most probable."

The time at which these octohedra occur in rheumatic urine varies considerably. They frequently exhibit themselves in the acute disease.

An elaborate series of experiments is given, in which the effects of diet and exercise on the deposit were analysed. These must be examined in the original, but it may be as well to state, as among the inferences from them, that while exercise is moderate, the difference of specific gravity of the urine, following the use of animal food only, or of animal food and bread, is very small. When the exercise is increased, the specific gravity is considerably greater than when the diet is purely animal.

During one entire month, the person on whom these experiments were made, took no vegetable substance, except bread, and even this was wholly abstained from for eight days; nevertheless at the end of the month oxalate of lime appeared in large quantities.

XIV. *On obstructions of the branches of the pulmonary artery;* by James Paget, Esq. From the arrangement of the pulmonary arteries, between which there is no anastomosis, except in their capillaries and smallest branches, the author points out, as a necessary result, that whenever the flow of blood through any part of these capillaries is prevented, there must be a stagnation of the blood in all the branches from which these capillaries are derived. Under these circumstances, the blood coagulates in the vessels and goes through various changes.

The affections mentioned by Mr. Paget as liable to become complicated with such coagulation of the blood in the pulmonary vessels are pulmonary apoplexy, œdema of the lungs, pneumonia, and cases of medullary cancer or of softened scirrhus, in which the cancerous matter passes into the circulation, and is stopped in the lungs. Cases of the latter description are to be found related many years past; and the explanation of the appearances now given by Mr. Paget has long been the current one. The detection of such clots is not a matter of novelty in pneumonia, having been described by Grisolle and others. But cases where the original affection of the lung has been apoplexy or œdema have not, as far as we know, been hitherto noticed.

Besides this, Mr. Paget gives three cases in which obstruction appears to have occurred as a primary affection, independent of capillary accumulation; in one of them there was disease of the trunk of the pulmonary artery and its valves. These cases will not bear condensation; but we recommend their study in the original. This is a useful essay.

XV. *On the composition of the meconium, and of the vernix caseosa or lubricating matter of the new-born infant,* by Dr. Davy. Meconium exhibits under the microscope a confused mixture of globules, plates, and molecules. The globules are inferred to be mucous; the plates of two kinds, epithelial and cholesteric; the molecules fatty. A soluble material connected with these ingredients seems identical with the colouring and sapid matter of the bile.

The vernix caseosa is composed of tessellated epithelium and fat molecules.

M. Raspail taught that a portion of the meconium consists of intestinal villi; Dr. Davy has sought in vain for the appearances he describes, and believes him to have been deceived by the aspect in certain positions of plates of cholesterine.

The two substances must be regarded as excretory; the one from the liver, the other from the skin.

XVI. *On paracentesis thoracis, as a curative measure in empyema and inflammatory hydrothorax;* by Dr. Hamilton Roe.

XVII. *Account of a case of empyema, which recovered after repeated punctures of the pleural sac;* by Dr. Theophilus Thomson.

Both these papers will be noticed in a forthcoming article on Empyema.

XVIII. *Observations on the omental sacs, which are sometimes found in strangulated herniæ, completely enveloping the intestine; with cases and dissec-*

*tions. To which has been added a table of all the strangulated herniæ operated on at St. George's Hospital in* 1842-3; by P. Hewett, Esq. The writer of these remarks describes a condition which has been rarely met with, and is very imperfectly noticed by Sir Astley Cooper and other systematic authors. The condition is that in which the intestine is "contained in a complete sac formed by the omentum, which it is absolutely necessary to divide, to reach the gut." Such sacs were found in four of thirty-four cases of operation for strangulated hernia in the course of two years—two were femoral, one inguinal, one umbilical. Richter referred the formation of these sacs to firm agglutination of the margins of the omentum which has surrounded the bowel. The two following explanations have been added: "1st, the gut, completely enveloped by the omentum, passes through the ring, and the omentum becomes attached to the circumference of the neck of the hernial sac. 2d, an epiplocele takes place, and the portion of omentum which is protruded becomes altered in structure, and its folds firmly united to each other by effused lymph. But the folds into which the omentum has been drawn *within* the abdomen, by the escape of a portion of it *without* that cavity, may not be agglutinated; so that spaces are left into which a knuckle of intestine may force its way, and thus form an intestinal, superadded to an omental, hernia."

These omental sacs may attain great size; and alterations of structure, leading to thickening, &c., may so affect the state of the parts as to become the source of great difficulties in the operation.

The omental sac may either lie loose in the cavity of the hernial sac, or the two sacs may have contracted adhesions with each other.

In cases where the hernial sac *appears* to contain thickened omentum only without intestine, the absence of the latter should not be made matter of too hasty conclusion; but a longitudinal incision practised in the whole length of the thickened omentum, if the fact of the non-inclusion of intestine be not otherwise satisfactorily ascertainable.

The intestine contained in the omental sacs described was adherent to their internal surface in one instance, non-adherent in the other.

The neck of an omental sac may become the sole cause of strangulation. One of the cases well illustrates this fact: Mr. Hawkins was obliged to divide the neck of the omental sac, before the gut could be reduced. Such cases, the writer very correctly observes, afford a striking argument against the practice of reducing a hernia without opening the hernial sac; had the practice been followed in this case, strangulation would have continued after, as before, the operation.

Free hemorrhage may occur from division of the arteries of the omental sac; precaution should be taken, as far as possible, to prevent the entry of the effused blood into the abdominal cavity.

This paper throws light upon an interesting point of practice.

XIX. *Account of a case of a dissecting aneurism of the aorta, innominata, and right carotid arteries, giving rise to suppression of urine and white softening of the brain;* by Dr. R. B. Todd. A stout gentleman, aged 37, fainted at dinner, quickly recovered, but then complained of abdominal pain, nausea, and pain in the back. The abdomen was now tense and swollen, but not very tender to pressure, the pulse extremely feeble and irregular.

The patient vomited, and had his pain relieved by bran poultices. The next day violent lumbar pain, extending to the groin and testicles, existed, urine scanty, no sickness, bowels open, limbs with natural power, drowsiness. Dr. Todd saw the patient seventy-two hours after the first attack: no urine had passed for forty-eight hours; stupor, but ability to answer questions rationally when roused; right pupil larger than left; paralysis of left side of the face, deviation of tongue to right; left upper and lower limbs slightly paralysed; twitchings; no reflex action excitable; these paralytic symptoms had come on suddenly a few hours before Dr. Todd's visit; no urine in bladder; no nausea or vomiting; pulse small and feeble on right side, full on left; bellows' murmur with first sound at the base of the heart and in the direction of the innominata. The patient's health had been good up to the time of the attack, he hunted, and eat—as hunters only can eat; he stated that "he had always felt some difficulty on the right side, as if he could not breathe as freely as on the left." The "respiratory murmur on the right side was not so loud as that on the left, but the difference was not more remarkable than is frequently observed in the most healthy persons." It occurs to us to inquire of Dr. Todd how often he has "in perfectly-healthy" chests detected an excess of force in the respiratory murmur on the *left* side? had he not held the opinions here inferred, his diagnosis might have been nearer the mark,—at all events he would have been led to look for a cause of the phenomenon. The urinary secretion was restored in a few days—urine albuminous; the cerebral symptoms eventually seemed to have somewhat yielded; but at twelve o'clock at night of the eleventh day of the attack, "just as he had finished drinking, he was seized with a slight convulsion, and fell back dead." The centrum ovale on the right side was "worm eaten in patches," the patches colourless, and softened to such a degree that the cerebral substance in them floated away under a stream of water; there was no pus or blood in them; the whole right hemisphere was particularly anemic, the left natural. The arteries of the affected hemisphere free from disease. The pericardium was distended with blood, which had escaped through a rent large enough to admit a goose-quill, situate an inch above the orifice of the vessel. The outer coat of the arch of the aorta was separated for some extent from the middle by an accumulation of blood, ulceration of an ætheromatous patch having opened a passage through the two inner coats for the blood. "This separation of the coats proceeded along the posterior surface and convex edge of the arch, involving the innominata artery, and to a partial extent the left carotid and subclavian. The middle coat of the aorta was split into two layers, between which the blood formed for itself a new channel, down to the abdominal aorta, in which it was evident, from the presence of recent coagula in it, that blood had recently flowed. A new channel was likewise formed by the splitting of the middle coat of the innominata on its outer and posterior part. This channel extended up to the lower part of the common carotid artery, and the blood accumulated there to such an extent as to obliterate the course of the artery completely, and effectually to stop the ascent of the blood into it. Two channels were thus found in the innominata; one leading to the carotid, which was formed by the splitting of the middle coat; the other the natural one, much diminished in size, through which the subclavian was supplied." The new channel in the aorta, the reporter of the case supposes, must have extended sufficiently low to impede the flow of blood in the renal arteries; but the part was not examined. The liver was large, the kidneys said to have been in "the second stage of granular disease,"—nothing is stated concerning them, but that they were large and congested, and these states (albuminous urine existing even) do not suffice to prove that this was not simple nephritis; we do not deny the existence of the alleged disease, but the evidence should have been given. The fainting and pain in the back Dr. Todd ascribes to the tearing up of the aortic walls and sudden diversion from the brain of the quantity of blood supplied greatly by one carotid artery.

Dr. Todd thinks the case a very conclusive one in favour of the doctrine which ascribes white softening of the brain to a process analogous to senile gangrene. It is not possible, from Dr. Todd's anatomical description, to form a very precise judgment concerning the date or stage of progress of the softening observed in this individual case; nor does he himself make any direct statement as to the period he may have supposed it to have existed. But he seems to consider that it was produced as a secondary lesion during the fatal illness described: the patient was healthy, and had been recently "twice passed as a healthy assurable life, by men of great discrimination." But men of great discrimination, whose minds have had no special bias towards the study of cerebral disease, may, without great strain of imagination, be supposed to allow a case of slight latent softening of the brain to slip through their fingers, and yet have no slur cast upon that discrimination. Was this case one of this description?—probably (but by no means certainly) not.

The narrative of the case is very creditable to its author.

XX. *Case of aneurism of the external iliac, in which a ligature was applied to the common iliac artery;* by R. Hey, Esq. Mr. Hey tied this vessel for aneurism, rapidly enlarging, of the left external iliac; the wound healed, and the ligature came away on the twenty-eighth day after the operation, and about fifty days after it the patient had recovered his usual health. The operator refers to the following circumstances as affording much anxiety to the medical attendants. The patient complained of a constant sense of distension of the bowels accompanied with violent spasm, especially when the bowels were moved: in a word, enormous distension of the rectum with hardened fæces took place, alarming symptoms were induced, but relieved at once by mechanical removal of the contents of the rectum. The pressure of the aneurismal sac on the colon prevented the contents of that viscus from descending into the rectum, thereby causing a gradual accumulation which was set free by the absorption of the contents of the sac.

"This is the first case which has occurred in this country in which aneurism of its branches has been cured by tying the common iliac artery."

XXI. *Two cases of tubular expectoration from the bronchi in the adult;* by Dr. Reid. Two cases of pseudo-membranous bronchitis, in which the plastic matter formed hollow casts of some of the bronchial tubes, are described under the above title. The narrations scarcely bear condensation, and we prefer referring the reader to the original; in his exordium Dr. Reid very palpably exaggerates the rarity (which is no doubt unquestionable) of such cases. "The disease is of too rare occurrence," observes the writer, to enable us to pronounce whether it is hereditary or not. Still, the fact of two brothers, of different callings and living in different places, having so rare a disease, tends very strongly to show, that it depends on some inherited peculiarity of constitution. The more severe complaint, which may be considered in some respects analogous to it, and to which the French authors have applied the term of "Angine couenneuse," appears to be so occasionally. Thus, by Bretonneau's account, the Empress Josephine died from the effects of this disease; her daughter, Hortense, was for some time subject to it; the son of the latter died from an attack of croup; whilst her nephew, the Duke of Leuchtenberg, consort of the Queen of Portugal, fell a victim, at a later period, to a complaint of a similar nature.

XXII. *A tabular view of the seat of tubercle, in* 180 *cases of tubercles of the lungs in children, with remarks on pulmonary phthisis in the young subject;* by P. Hennis Green, M.B. The writer's design in this paper is to indicate a few of the peculiarities which distinguish infantile consumption from the phthisis of adults, rather than to give any detailed history of phthisis, as it occurs in the young subject.

The paper contains a tabular view of the seat of tubercle in 180 cases of the disease occurring in males, aged from two to fifteen years.

The main character, it is stated, which distinguishes the phthisis of children from that of adults, is that in the former the tubercular deposit occupies a much larger surface of the lung, is more rapidly secreted, and is complicated with tubercular disease of other organs, more frequently than in the adult. Hence children sink under the affection earlier than grown persons, and the disease, from the diffusion of the tuberculous matter, is with greater difficulty to be diagnosticated. Bronchial phthisis is peculiar to the child.

Miliary tubercles, yellow infiltration, and the common yellow crude tubercle, are the forms under which the disease exhibits itself. "Yellow infiltration" ought to have been, but is not, described.

Crude tubercle in the child, as in the adult, more frequently occupies the superior lobes than any other part of the lung. Of 112 cases, the disease existed as follows, in respect of the lung attacked:

| | |
|---|---|
| Both lungs . . . . | 101 |
| Right lung only . . . . | 3 |
| Left lung only . . . . | 8 |

In these 112 cases the deposit was confined to the bronchial glands in 12 cases; to the pulmonary tissue in 12. In 68 other cases the bronchial glands alone were tubercular, and in 1 the pulmonary tissue alone. In 3 cases tubercle existed in the cavities of the head or abdomen, and had not formed in the chest. This shows, as the writer observes, that M. Louis' law does not apply to children; but M. Louis never so applied it.

Cavities existed in 31 of the 112 cases; on the right side in 12, the left in 11, and both sides in 8 cases. It is a point of importance that in children under five years of age the excavation is generally seated in the lower or middle lobes, and is almost always confined to one side of the chest. As the child approaches the age of twelve or fourteen years, the cavern occupies the upper lobes, and is often found in both lungs, as in the adult.

Caverns are of two kinds; one precisely similar to that occurring in the grown person; the other variety (peculiar to the child, and most common before three years of age,) is produced by the process of softening taking place in the midst of yellow infiltration.

Dr. Green gives a very interesting parallel between adult and infantile phthisis, in respect of the frequency with which other organs besides the lungs are implicated in the tuberculous disease. We shall throw this parallel into a tabular form.

| ADULT. (Observed by Louis.) | | CHILD. (Observed by Green.) |
|---|---|---|
| *Tubercles* in | | |
| Brain or membranes | 1/358 of the cases | 1/9 of the cases. |
| Bronchial glands | 1/5 ,, | 100/112 ,, |
| Mesenteric glands | 1/5 ,, | 1/2 ,, |
| Liver | 1/179 ,, | 1/9 ,, |
| Kidneys | 1/34 ,, | 1/18 ,, |
| *Ulcerations* in | | |
| Larynx | 1/4 ,, | 1/112 ,, |
| Bowels | 5/6 ,, | 1/7 ,, |

The value of this parallel needs not to be insisted upon.

Two forms of this disease must, according to the author, be separately considered in respect of symptoms. In the one the functional phenomena are the same as in the adult phthisis (this occurs in children aged from ten to fourteen); this form the author correctly considers it unnecessary to describe. In the other class of cases, "the complaint is commonly acute from the commencement, and is accompanied by various signs of the tubercular diathesis. The child has irregular accesses of fever, with heat of skin, acceleration of the pulse, and slight flushing of the cheeks, while the rest of the face is pale and haggard. The tongue is often furred, and red at the edges; the abdomen sometimes tumid, and the bowels irregular; the child may complain of headache and want of sleep; the appetite fails, and emaciation soon sets in. The patient is now seized with a small, dry cough, accompanied by oppression and acceleration of the breathing; there is no expectoration; on examining the chest, the physical signs are few and uncertain. When the miliary tubercles, or granulations, are sufficiently numerous, and collected in the upper lobes, we may perceive a certain roughness in the respiratory murmur, and some prolongation of the expiratory sound; but these signs are frequently masked, either by the existence of other lesions in the chest, or by the tubercular deposit being seated in the lower or middle lobes. The presence of tuberculous infiltration is indicated by feebleness or absence of the respiratory murmur, with some dulness on percussion; but these signs are common to chronic pneumonia."

But even where cavities exist, physical signs are often completely absent, when the child is under five years of age. The importance of the fact is sufficiently obvious, no matter "whether this absence of cavernous signs depends on the seat of the cavity, on its anfractuous formation, or (as

MM. Rilliet and Barthez have suggested) on the small caliber of the bronchial tubes in very young children."

The cough occasionally occurs in paroxysms somewhat resembling those of hooping-cough. In such cases, the author thinks there is reason to believe that the paroxysmal character depends on pressure by enlarged bronchial glands.

The respiration, with the advance of the disease, *may* reach 80 in a minute ; but if the affection run a chronic course, the breathing is seldom much accelerated.

Dr. Green relates a striking example of the well-known fact that young children swallow everything that comes from the lungs. A child, two years old, died suddenly from the rupture of a blood-vessel in a cavity. A very small quantity of blood had been discharged through the mouth ; but after death the stomach and duodenum were found full of enormous clots of blood.

The writer observed hemoptysis five times in the 112 cases,—in two instances sufficiently abundant to cause sudden death. His experience is somewhat peculiar here: the extraordinary rarity of hemoptysis in children is universally recognized. And it is to be observed that in Dr. Green's five cases, the patients were all aged upwards of nine years.

Dr. Green adds nothing to our existing means of distinguishing acute phthisis from lobular pneumonia.

The bronchial glands were more or less extensively tubercularized in 100 of 112 cases. In a few only of these cases, however, were the glands sufficiently enlarged to produce symptoms through their mechanical effects, or by communicating with caverns in the lungs, or with the bronchi ; to such cases, the writer justly observes, the term bronchial phthisis should be confined. Various symptoms dependant on pressure of the great vessels, œsophagus, and trachea, may be observed. The diagnosis of this form of phthisis in children must, it is said, be based upon the absence of the physical sign of tubercles in the lungs, in cases where the rational symptoms of phthisis are present. But, as we have already seen that the presence of tubercles in the lungs, and even of caverns, may be masked by various circumstances, it does not appear that the principle here laid down may always, or even commonly, lead to a very satisfactory diagnosis.

This is a truly valuable paper.

XXIII. *Case of a tumour in the right hypochondrium occurring after injury, from which a large quantity of fluid resembling bile was repeatedly withdrawn by the operation of tapping;* by W. B. Barlow, Esq. A strong healthy man, aged 54, injured himself by lifting a heavy ladder ; he complained of severe pain in the region of the liver. Two days after, the motions were nearly white, and the urine dark, as of a jaundiced person. On the seventeenth day, a swelling the size of a walnut was observed over the region of the liver. About six weeks after the accident, no bile having yet passed per anum, and the swelling being greatly increased in size, it was tapped, and seven quarts of a fluid composed in by far the greatest part of bile, drawn off. Subsequently six and a half quarts, seven quarts, six quarts, four and a half quarts, and three pints of a similar fluid were removed at different periods. The bile ultimately resumed its proper course, and in about four months after the accident the man was convalescent.

XXIV. *Peculiar case of gelatiniform cancer, in which nearly all the organs of the body contained colloid tumours, with the appearances on dissection;* by J. C. Warren, M.D. (U. S.) A painter, aged 25, applied to Dr. Warren in May, 1840, on account of a tumour on the right side of the neck: it had existed for a year and a half. He died on the 21st of the following September. Among the post-mortem appearances were the following: The surface of the body was studded with subcutaneous tumours, "composed of small granulations, constituted by sacs containing a substance which appeared at first view to be wholly gelatinous, but which, on being divided, discharged a small quantity of viscid fluid. The colour was a mixed gray and red; they were slightly transparent; in consistence they were friable: most of them were connected with small veins, and had a vascular tissue upon their outside." Similar tumours existed in the thyroid gland, diploe, muscles, mediastinum, muscular substance and cavities of the heart, lungs, pancreas, kidneys and testes. The tumour in the neck resembled a scrofulous enlargement of glands, as likewise did various diseased lymphatic and mesenteric glands. The liver contained scirrhous nodules; the absorbent vessels of the surface of the abdomen contained a matter having some of the characters of encephaloid. The general dissemination of these little semi-transparent tumours, and their losing their transparency in alcohol, induced Dr. Warren to doubt whether they were really specimens of colloid cancer, or of some peculiar and distinct formation. The case is a valuable contribution to, and deserves to be specially studied by those engaged in the pursuits of, pathological anatomy.

XXV. *An account of the examination of a cyst containing seminal fluid;* by James Paget, Esq. Guided by certain observations described in this paper, Mr. Paget considers that "the most probable explanation of the occurrence of spermatozoa in the fluid of cysts connected with the testicle, seems to be that certain cysts, seated near the organ which naturally secretes the materials for semen, may possess a power of secreting a similar fluid." The grounds of this hypothesis will be found in the original.

XXVI. *Some statistical records of the progress of the Asiatic cholera over the globe;* by W. J. Merriman, M.D. This paper, though highly creditable to the industry of its author, does not require, in the present condition of public health, to be particularly noticed.

XXVII. *Case of necrosis of the lower jaw, recovered from without deformity;* by W. Sharp, Esq. The title of this paper is quite as communicative as the narrative it introduces.

XXVIII. *Remarks on the pathology of mollities ossium, with cases;* by S. Solly, Esq. Mr. Solly relates, and illustrates with lithographs, two cases of osteomalacia; he thus relates his views concerning the nature of the affection: "After a careful consideration of all the facts, but especially by comparing the appearances after death, with the symptoms during life of this awful disease, I am led to believe that it is of an inflammatory character. That it commences with a morbid action of the blood-vessels, which gives rise to that severe pain in the limbs invariably attendant on this disease, but more especially in its commencement, and exhibits itself after death by an arterial redness of the part. The absorbent vessels are at the same time unnaturally excited, and the earthy matter of the bone is absorbed, and thrown out by the kidneys in the urine, which excretion is sometimes so abundant, as we have seen in the last case, that it clogs up the calices and pelvis of the kidney, and forms there a solid calculus . . . . The place of the phosphate of lime in the bones is supplied by that morbid secretion of red grumous matter, which has been so universally found in this disease." The author adds little to the facts established by Miescher, Gluge, Müller, and G. O. Rees (inter alios): as regards the theory of inflammation, it is wholly hypothetical,—"arterial redness" does not prove the presence of inflammation, and even if it did, where is the evidence that the inflammation was otherwise than secondary in the sequence of events?

XXIX. *Case of fistulous communication between the intestinum ileum and urinary bladder, simulating stone in the bladder*; by W. C. Worthington, Esq. It seems rather difficult to imagine how a "communication" can "simulate" a "stone;" but this is a mere verbal difficulty. However, in point of fact, the symptoms existing in the case do not strike us as those of calculus, but simply of chronic cystitis. What was the design in submitting a meagre old woman, aged 65, "suffering from an obscure abdominal affection," to a "mild mercurial course?" When will the indiscriminate abuse of mercury cease to be a stain on English practice?

XXX. *Observations on the recorded cases of operations for the extraction of ovarian tumours*; by B. Phillips, Esq. Here is the "conclusion, where nothing is concluded." At least it is difficult to extract from the writer's pages any precise inference as to admissibility of this operation. This however, arises not from Mr. Phillips's deficiency, but from the nature of the case he has to examine. "The experience we possess," he says, "justifies us in the expectation that in forty-four cases out of 100, the tumour may be extracted, and life saved; but at the same time it cannot be concealed that out of the eighty-one operations to which we have referred, thirty-two died, and that soon, in fact in a very few days. Whether these results justify a medical man in recommending a patient to submit to the operation, is a question which will probably be decided differently by different men, perhaps in neither case upon the merits."

It is to be observed that all unfortunate cases have not been chronicled; Mr. Phillips makes allusion to five unrecorded operations, three of which at least terminated by death.

The tables accompanying this paper are valuable and judiciously put together, like everything that comes from the pen of Mr. Phillips.

